# Dataset for detecting and characterizing Arab computation propaganda on X

**DOI:** 10.1016/j.dib.2024.110089

**Published:** 2024-01-23

**Authors:** Bodor Moheel Almotairy, Manal Abdullah, Dimah Hussein Alahmadi

**Affiliations:** Department of Information systems, Faculty of Computing and Information Technology, King Abdulaziz University, Jeddah, Saudi Arabia

**Keywords:** Arabic computation propaganda, Disinformation, Propagandists’ characteristics, Social media, Propaganda classification

## Abstract

Arab nations are greatly influenced by computational propaganda. Detecting Arab computational propaganda has become a trending topic in social media research. Despite all the efforts made, the definitive definition of a propagandistic characteristic is still not clear. Additionally, the earlier datasets were acquired and labelled for a specific study but were neglected thereafter. As a result, researchers are unable to assess whether the proposed AI detectors can be generalized or not. There is a lack of real ground truth, either to characterize Arab propagandist behaviours or evaluate the new proposed detectors. The provided dataset aims to demonstrate the value of characterizing Arab computational propaganda on X (Twitter) to close the research gap. It is prepared using a scientific approach to guarantee data quality. To ensure the quality of the data, the propagandist users’ data was requested from the X Transparency center. Although the data released by X relates to propagandist users, at their level, the tweets were not classified as propaganda or not. Usually, propagandists mix propaganda and non-propaganda tweets to hide their identities. Therefore, three journalist volunteers were employed to label 2100 tweets for either propaganda or not and then label the propagandist tweet according to the propaganda technique used. The dataset covers sports and banking issues. As a result, the dataset consists of 16,355,558 tweets with their meta data from propagandist users in 2019. Plus, 2100 propagandists labelled tweets. The propagandist's dataset helps the research community apply supervised and unsupervised machine learning and deep learning algorithms to classify the credibility of Arab tweets and users. On the other hand, this paper suggests looking at behaviour rather than content to distinguish propaganda communication. The datasets enable deep non-textual analysis to investigate the main characteristics of Arab computational propaganda on X.

Specifications Table

**Table:** 

Subject	Computer Science, Data Science, Social Science.
Specific subject area	Text Classification, Data analysis, Social media, Arab computational propaganda detection, Arab computational propaganda characterizing, Social Network Analysis.
Data format	Raw, Filtered.
Type of data	Databases include JSON files of propaganda data on X.
Data collection	The propogandist data was requested from the Twitter (X) Transparency center^1^, and a sample of it was annotated with the help of three journalists’ volunteers.
Data source location	https://twitter.com, Saudi Arabia.
Data accessibility	Repository name: Mendeley Data: “Arab Computational Propaganda on X (Twitter)” Data identification number: DOI: 10.17632/58mttpbc7x.3 Direct URL to data: https://data.mendeley.com/datasets/58mttpbc7x/3

1 Information Operations - X Transparency Center (https://transparency.twitter.com/en/reports/moderation-research.html).

## 1 Value of the Data

• The unlabeled propagandist dataset is large and has a diverse collection of tweets and meta data. We can combine it with the labeled propaganda dataset using a semi-supervised learning strategy. This may decrease the need for manual annotation by allowing the AI model to learn from both the characteristics and the patterns of the data.
• The labeled propagandist dataset may be used to train supervised and unsupervised deep learning and machine learning algorithms to detect Arab computational propaganda.
• The labeled propagandist dataset enables the development and validation of new data-driven techniques, such as Natural Language Processing (NLP), to extract propaganda-related techniques from Tweets.
• The dataset helps researchers conduct a deep non-textual analysis and data visualization of two different communities to investigate the key features and patterns that can differentiate between propagandists and non-propagandists, regardless of their goal.
• The features and patterns that can be discovered from the dataset help researchers validate new non-textual or multi-modal models to detect propaganda on X.
• Researchers can use the discovered features after analysing the dataset to annotate the huge existing propaganda tweets using the recently proposed programmatic or weak supervision methods such as active learning or transfer learning.
• Techniques for unsupervised learning can be used to find hidden representations, clusters, or structures in the data and improve performance on subsequent tasks.

## 2 Data Description

**Fig. 1:**
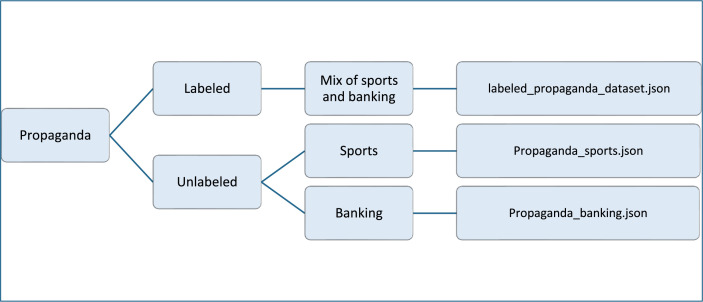
Datasets classification.

**Fig. 2:**
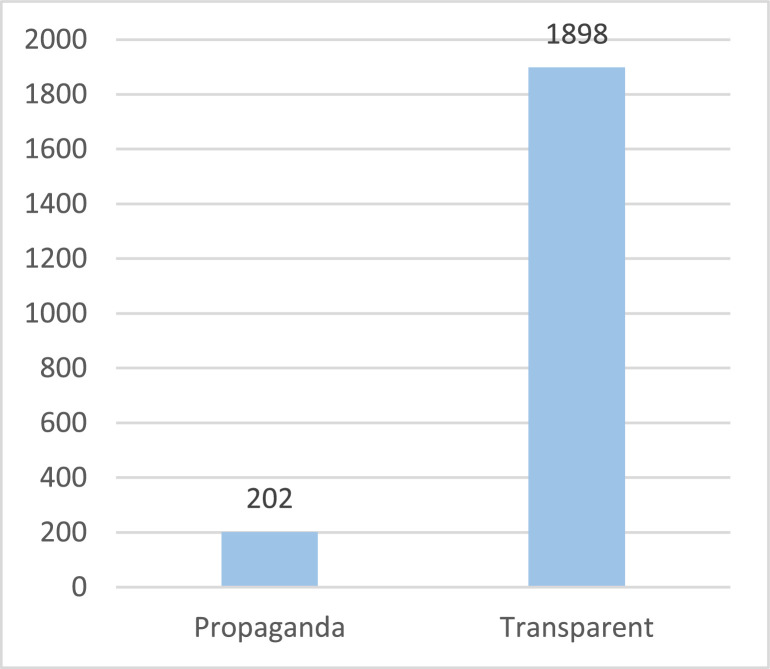
The number of propaganda and transparent tweets.

**Fig. 3:**
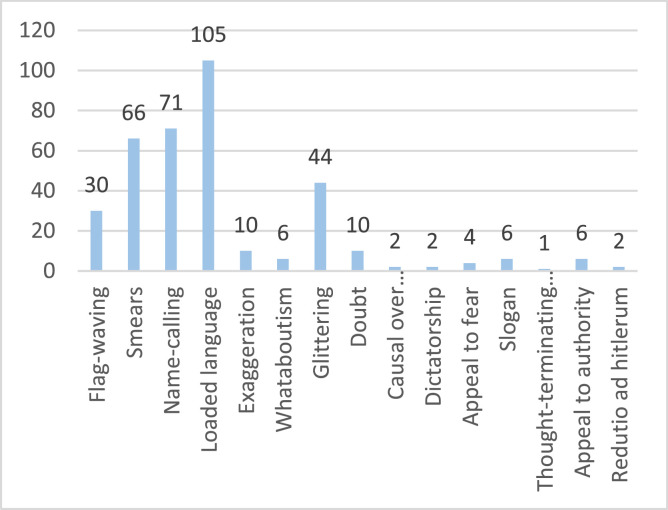
The number of each technique in the dataset.

The database includes three datasets; Fig. 1 illustrates dataset splitting. There are propaganda data for sports and banking topics. Although X released propagandists’ data that focuses on malicious users who spread propaganda, at their level, the tweets were not classified as propaganda or not. Usually, propagandists spread propaganda and non-propaganda tweets to hide their identities. Therefore, there is a need to classify their tweets as propaganda or not. Because the datasets are very large, we annotated a sample of 2100 tweets. The annotators annotated the tweets as propaganda or transparent (non-propaganda). Then the propagandists’ tweets were annotated based on the used propaganda techniques. The distribution of the data classes is shown in Fig. 2, which the distribution of techniques used is shown in Fig. 3. Table 1 shows the size of each dataset.

**Table 1:** Size of the datasets.

Annotation	Topic	Tweet	Users
Unlabeled	Sport	14,778,400	4779
	Banking	1577,158	5196
Labeled	General	2100	579

All the datasets are saved in JSON format. Based on the X’ Developer Agreement and Policy,^1^ we removed the user screen name, user display name, user profile description, and user location to hide their identity. Table 2 shows the features in each dataset. In the “Format” column, R indicates that the features are raw, while F indicates that the features are filtered. For example, “hashtags” is the raw data, while “num_hashtags” includes the total number of hashtags calculated by the authors. The same thing was applied to “URL” with “num_urls” and “num_user_mentions” with “user_mentions”. Finally, the features “label” and “propaganda tech” were applied only to the labelled propaganda datasets, which were annotated by experts as explained in Section 3.4.

**Table 2:** Features of datasets.

Feature	Description	Format	Propaganda		
			Sports & Bank	Sports	Banking
follower_count	Total number of accounts that follow the user	R	✓	✓	✓
following_count	Total number of accounts that followed by the user	R	✓	✓	✓
account_creation_date	Account's creation date	R	✓	✓	✓
tweet_text	Tweet's content	R	✓	✓	✓
is_retweet	Bool variable indicates that this tweet is being retweeted	R	✓	✓	✓
quote_count	Total number of tweets that mention this tweet	R	✓	✓	✓
reply_count	Total number of tweets that replied to this tweet	R	✓	✓	✓
like_count	The total number of likes garnered by this tweet	R	✓	✓	✓
retweet_count	Total number of times this tweet was retweeted	R	✓	✓	✓
num_hashtags	Total number of hashtags embedded in this tweet	F	✓	✗	✗
hashtags	List of hashtags that were embedded in this tweet	R	✗	✓	✓
num_urls	Total number URLs that were utilized in this tweet	F	✓	✗	✗
URL	List of URLs that was utilized in this tweet	R	✗	✓	✓
num_user_mentions	Total number of usernames mentioned in this tweet	F	✓	✗	✗
user_mentions	List of usernames mentioned in this tweet	R	✗	✓	✓
Labels	Class of the tweet: propaganda or transparency	F	✓	✗	✗
propaganda_tech	Propaganda techniques used in the tweet	F	✓	✗	✗

## 3 Experimental Design, Materials and Methods

**Fig. 4:**
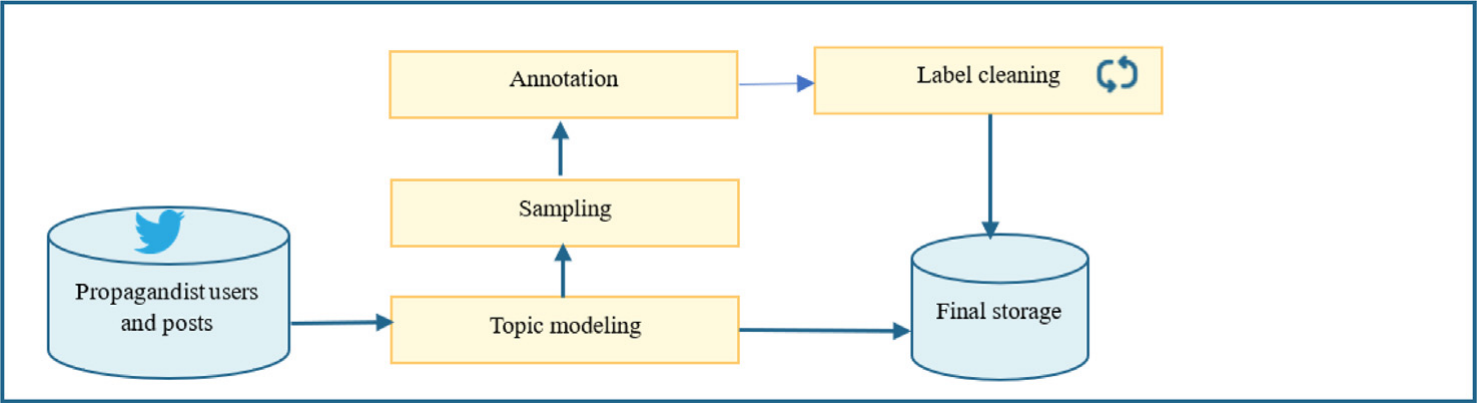
Data collection framework.

Investigating propagandist behaviors on X requires data about their profiles, tweets, and activities. As shown in Fig. 4, the process starts by requesting propagandist data from X, which includes data about propagandists’ users and their tweets. The main topics will be detected from the propagandists’ tweets. Then, a sample from each topic will be taken, annotated by experts, and then refined using a confident learning method. At the same time, the data on two topics (banking and sports) will be stored with their metadata. The next subsections describe the data collection procedures in detail.

### 3.1 Requesting propagandist data

X provides publicly available archives of tweets through the X transparency website, which includes timely disclosure of information regarding organizations attempting to use X to manipulate public opinion. The selected propagandist dataset was published in 2019- 2020. It contains 5929 Saudi accounts and 50 M tweets on general topics. The dataset was published in two files: one includes the users’ fields, while the other includes the tweets’ fields. The users’ fields file includes 10 features about the users’ meta data: user id, display name, screen name, location, profile description, follower counts, following counts, account creation date, and account language. The tweet field file includes twenty features in addition to all the features mentioned in the user's file: tweet id, tweet language, tweet text, tweeting time, tweet client name, latitude and longitude of geolocation if available, the list of hashtags that were used in this tweet, the list of URLs that were utilized in the tweet, the list of usernames that were mentioned in the tweet, and the total number of tweets that mention, reply, like, and retweet this tweet. The other six features are about the tweet's originality. They are a bool variable to indicate if this tweet is a retweet or not; the user id of the original user to whom this tweet is a reply or retweet; and finally, the id of the original tweet to which this tweet is a reply, quote, or retweet. *We filtered the unneeded data and kept only the data that could be used to distinguish the propagandists, as shown in* Table 2*.*

### 3.2 Extracting propagandists' topics

One of the famous efforts in the topic modelling is using FastText.^2^ It is unsupervised model to represent the language's hidden information in the text as vectors and then implement K-means clustering to group texts into topics [1]. FastText is an open-source package developed by Facebook AI Research (FAIR). It enables the development of supervised and unsupervised learning algorithms for generating word vector representations. Furthermore, it guarantees that even rare words will have the proper vector embeddings. On the other hand, K-means clustering is a popular unsupervised machine learning approach. It is used to group together relevant data points [2]. It helps to discover patterns in data as well as organize written content into themes [2]. Applying FastText and K-means clustering consecutively helps to identify patterns in the data and group similar text documents together. The next subsections describe the topic modelling steps.

#### 3.2.1 Data cleaning step

All the null values, non-Arabic texts, URLs, punctuation marks, whitespace, and new lines were removed. Cleaning the Arabic language is a vital step, as Arabic is an inflectional language. For lemmatization, Farasa Library (Arabic segmentation) was used.^3^ Normalization is applied to standardize the shape of Arabic words and letters so that they may be expressed in one form without compromising the meaning of the phrase [3]. The dataset is normalized using Python's Tashaphyne module.^4^ It supports light stemming (removing prefixes and suffixes) and offers all conceivable segmentations.

#### 3.2.2 Text vectorizing step

A neural network method called a Skip-gram model [4] was used to represent the text vectors. It predicts the words in the context given a target word [4]. The Skip-gram model has the advantage of being able to produce high-quality word embeddings that can represent the semantic and syntactic links between words [4]. In our experiment, we used the default values of the parameters.

#### 3.2.3 Clustering step

Because of the dataset is huge, was used Mini-Batch K-Means.^5^ Mini-Batch K-Means is a K-Means clustering algorithm version uses smaller random batches of data rather than the complete dataset for each iteration. In this experiment the batch size was 216. An elbow approach was utilized to identify the ideal number of clusters, K [5].

**Fig. 5:**
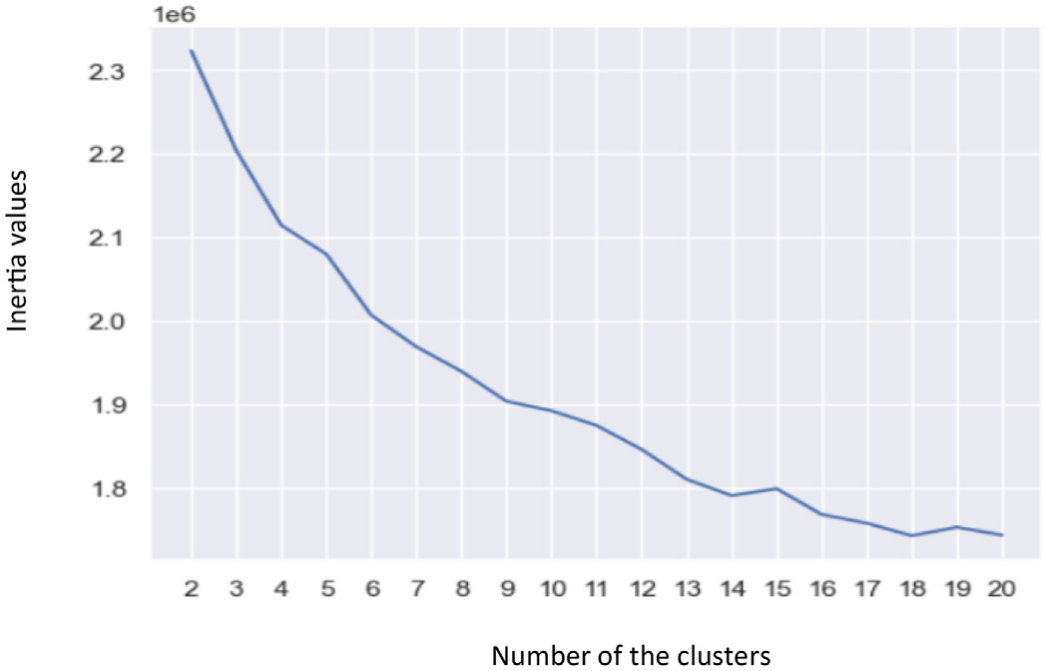
The number of the clusters versus their inertia values.

In the elbow approach, the value of k is continually iterated from *k* = 2 to *k* = *n* (n was set to 20) and calculated Inertia for each K. Inertia is a K-Means algorithm performance metric [6]. Fig. 5 plots a graph of k versus their inertia values. The graph was rapidly changed at a point and thus creating an elbow shape. The best value of K, or the ideal number of clusters, corresponds to this point. The “elbow” of this graph is *K* = 14.

#### 3.2.4 Main topics

**Fig. 6:**
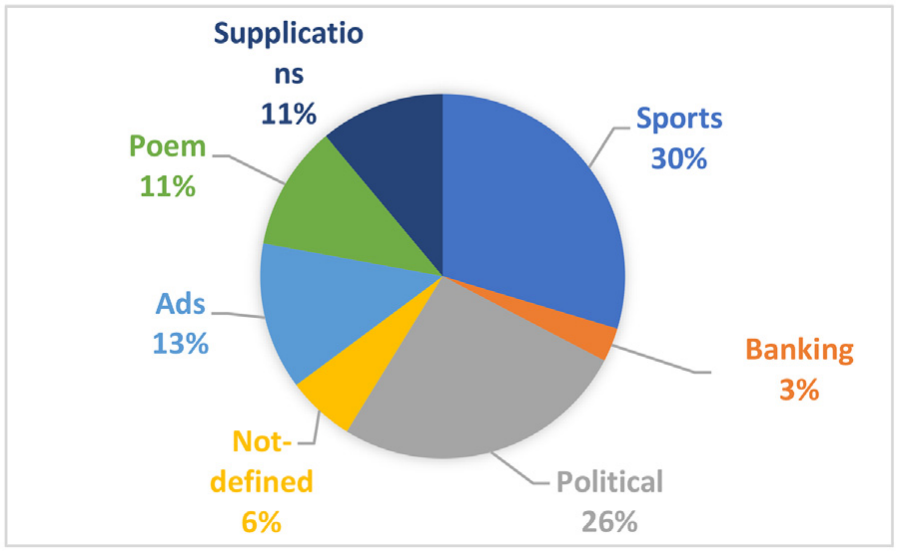
The percentage of each topic in the dataset.

With the help of a volunteer journalist,^6^^,^^7^^,^^8^ the main topics of the clusters were identified as follows: One political cluster includes tweets about countries issues and some political figures. Two sports clusters include tweets about clubs and players in Saudi football. Two social issue clusters include tweets about Saudi banking issues and strong objections to bank loans. Four clusters include tweets containing supplications. Three clusters include tweets containing poems. One cluster includes tweets containing different ads. One cluster includes tweets that contain only a few words that do not present any meaning. Fig. 6 shows the percentage of each topic in the dataset. There are three topics that can be investigated in the dataset: sports, politics, and banking topics. Therefore, this study focuses on sports and banking issues because political issues have been discussed in previous widely studies. Table 1 shows the size of propaganda datasets in sports and banking topics, while Table 2 shows the features in each dataset.

### 3.3 Annotating propagandists' tweet

We sampled the data from all the topics using simple random sampling. Random sampling is considered the more effective sampling method to obtain more representative sample X data [7]. Three journalists participated in the annotation processes.^9^^,^^10^^,^^11^ The annotation was conducted in two phases*:*

• Training phase: we had two meetings with a member of the Oxford Internet Institute.^12^ We discussed the propaganda techniques and how we can adopt them on the Arabic tweets [8]. Then, we gave the annotators explicit annotation guidelines with examples, and the annotators were asked to annotate a sample of tweets. After that, our team revised their annotation and offered commentary.
• Annotation phase: the annotation was conducted in steps. First, two annotators individually annotate the same tweet. Next, they met with one consolidator to debate each instance and come up with the final annotations. The second step is to make sure that the annotation is discussed by all annotators.

### 3.4 Finding and learning labels errors

Often, there are labelling mistakes that come from the annotators themselves, especially when the concept is complex, not clear, and depends largely on the annotator's understanding, as in our case. These are incredibly hard issues to track manually. So, we utilized AI to methodically enhance the data labels. We applied a confident learning method for this purpose [9]. Cleanlab,^13^ is a new confident learning method that can calculates errors calculates errors in the dataset using statistics derived from model probability predictions. The process of cleaning the labels was as follows: First, we converted the raw data to a format that allowed us to use ML. Then, we trained a logistic regression model on the formatted dataset. A K-fold technique was used to compute the probability predictions of each data point. Cleanlab was applied to detect label issues based on the computed probability. The annotators have fixed the label errors and trained the logistic regression model again on the enhanced dataset. We used this reasoning repeatedly to detect label errors, correct them, and train the model with the updated, ostensibly higher-quality labels. We found a total of 293 label errors in the 15 iterations. In each iteration, the annotation team held a decision-making meeting to define the propaganda techniques in social media. Finally, to make sure that the model can predict propaganda correctly based on the datasets, we trained AraBert.^14^ The prediction performance achieved 98 % precision in only 2 epochs.

To define propaganda techniques in social media, we mainly consider the definition of propaganda: “Propaganda aims at influencing people's mindset with the purpose of advancing a specific agenda” [10]. Based on this definition, we consider all tweets to be transparent, regardless of the technique used, as long as there is no intent to deceive the audience and change their mindset on a specific topic. At the same time, if a tweet seems to have the intent to change people's mindset but doesn't contain any of the reported 20 techniques, it will be regarded as transparent. As a result, ads for social media accounts, ads for products, supplications, and poems are regarded as transparent. Table 3 describes the final definition of the propaganda techniques used in social media to annotate the data.

**Table 3:** Computational propaganda techniques description.

No.	Techniques	Definition
1	Red Herring	Adding unrelated information to the topic under discussion to draw attention away from the ideas being presented.
2	Straw Man	When a comparable statement is used in lieu of an opponent's, the original proposition is disproved, for example, “The coach says that his team needs extra training more than focusing only on nutrition, so people claim that he does not care about proper nutrition for the players”.
3	Whataboutism	A tactic used to cast doubt on an opponent's position by accusing them of hypocrisy while avoiding openly challenging their claims, for example, “this coach never said what he thought”.
4	Causal Oversimplification	Assuming there is just one cause or explanation when there may be others, for example, “The team's performance has declined since this coach coached them. Therefore, this coach must be dispensed”. In this example the reasons for the team's decline were limited only to the coach.
5	Obfuscation, Intentional vagueness, Confusion	Using terms that are intentionally ambiguous to allow the audience to make their own interpretations, for example, “The secret organization manipulates the delicate balance of power from behind a curtain of secrecy”. In this example, although the statement uses obfuscation by utilizing complicated language, it does not give information on their activities or goals.
6	Appeal to authority	Appeal to authority stating that a proposition is true without providing any further proof, merely because an authority or expert on the subject indicated it was true. For example, according to renowned neuroscience specialist, Dr. Ahmed, meditation offers several advantages for mental health, so everyone should include it in their daily routines.
7	Black-and-white Fallacy, Dictatorship	Presenting two potential solutions as the only ones available when, in fact, there are more, for example,” Either our team captures the title, or the entire season is a total disaster. If we can't win, what's the point of playing?” Presenting one solution is considered a slogan, for example, “No to foreign tourism”.
8	Name calling or labelling	Labelling the target of the propaganda, whether present or hidden, as either something the target audience likes, appreciates, or fears, praises, or hates. For example, “The striker for the opposing side is nothing more than a dishonest cheater who dives to trick the referee at the first sign of contact”.
9	Loaded Language	Influencing the audience by using strong words and phrases that have significant emotional connotations (either good or negative), for example, “Our team was bravely fighting against the unjust and corrupt system.”
10	Exaggeration or Minimisation	It is amplifying things in an excessive manner, such as “the strongest,” suppressing things by making them seem smaller than reality, for example, “the worst,” or using conclusive words such as “undisputed”. This technique is strongly connected to labelling techniques.
11	Flag-waving	Using strong national feelings or any group feelings (such as gender or race) to promote an idea, for example, “The height of patriotism is represented by our national team; when they succeed, it's a victory for the entire nation”. Using only strong patriotic phrases without promoting any idea is considered a transparent tweet. For example, “We are the Arabs” or “Saudi women"
12	Doubt	Doubting the target's reliability or ability, whether they are present or hidden, for example: “I don't think they'll win.”
13	Appeal to fear	Injecting the fear and anxiety emotion into the audience with the intent to promote an idea, for example," If we don't enact this law, crime and disorder will take over our nation". Using fear words without any intention to influence the audience is considered transparent; for example, “We're all going to die”.
14	Slogans	a succinct and striking sentence that could include stereotypes and labels. Slogans frequently serve as emotive pleas, for example, “Become a part the Winning Team and Dominate the Game!”
15	Thought-terminating cliché	Brief and generic words or expressions that restrict discussion by giving simple answers to draw the reader's attention away from more significant topic, for example, “Have mercy and very thing will work out”
16	Bandwagon	Trying to convince the audience to take action because it is supported by a majority. The statement usually contains an expression about the topic followed by a call to action, for example, “Participate in the winning team's fan base and rejoice in their championship success.”
17	Reductio ad hitlerum	By implying that a particular action or thought is preferred by groups that the target audience despises and finds repugnant, one might persuade them to disagree with it. Any individual or idea having a bad connotation might be referred to in this way. The statement usually contains an expression about the topic followed by a call to action, for example, “This political party will destroy us; it has brought back the Nazi era; let us resist them.”
18	Repetition	Repeating the same message frequently, whether from the same malicious user or from a group of users (the digital army), Usually, people accept the statements that keep repeating.
19	Smears	It is an attempt to harm individuals' or groups' reputations by spreading false information about them, whether their names are declared or not. For example, Player X is said to have used performance-enhancing substances.
20	Glittering Generalities (Virtue)	Using the target audience's value system words such as happiness, hope, and peace to trick them into accepting something without considering the proof, for example: “Women's freedom is our demand.”

## 4 Limitations

1. The labelled dataset includes 2100 online posts, covering 15 propaganda techniques. The size and scope of the dataset (i.e., the number of covered technique categories) were limited by available resources, such as human annotators, and the allocated time for constructing the dataset.
2. The provided unlabelled dataset is restricted to Twitter data from Saudi Arabia, with a focus on banking and sports. Thus, it is decided that preliminary testing is required to apply our dataset to Twitter data from Arab countries with diverse topics.

## Ethics Statement

All data is distributed under the X Developer Agreement and Policy.^15^ The propaganda raw data are publicized for researchers on the X Transparency Center website, and all the propagandist accounts in the dataset are already blocked from X. Based on the policy, the datasets can be shared unless they are not used for any of the prohibited uses listed in the policy, such as tracking users, monitoring sensitive events, profiling individuals based on their health, political affiliation or beliefs, racial or ethnic origin, religious or philosophical affiliation or beliefs, or trade union membership. Moreover, we removed the tweet ID, user ID, user screen name, user display name, user location, and user profile description from the list to hide their identity.

## CRediT authorship contribution statement

**Bodor Moheel Almotairy:** Conceptualization, Methodology, Data curation, Visualization, Software, Investigation, Writing – original draft. **Manal Abdullah:** Conceptualization, Supervision, Writing – review & editing. **Dimah Hussein Alahmadi:** Conceptualization, Supervision, Writing – review & editing.

## Data Availability

Arab Computational Propaganda on X (Twitter) (Original data) (Mendeley Data).

## Footnotes

12 https://www.oii.ox.ac.uk/people/profiles/mona-elswah/.

## Footnotes

1 https://developer.twitter.com/en/developer-terms/agreement-and-policy.

2 https://github.com/facebookresearch/fastText.

3 https://farasa.qcri.org/segmentation/.

4 https://pypi.org/project/Tashaphyne/.

5 https://scikit-learn.org/stable/modules/generated/sklearn.cluster.MiniBatchKMeans.html.

6 https://twitter.com/qaburibrahim?s=11&t=c3Ln2hTg674xoeXohmncLw.

7 https://twitter.com/faisalalhmyane?s=11&t=c3Ln2hTg674xoeXohmncLw.

8 https://twitter.com/abdulaziz_ali?s=21&t=VKxoRqdza6UTnrWhF_fgIA.

9 https://twitter.com/qaburibrahim?s=11&t=c3Ln2hTg674xoeXohmncLw.

10 https://twitter.com/faisalalhmyane?s=11&t=c3Ln2hTg674xoeXohmncLw.

11 https://twitter.com/abdulaziz_ali?s=21&t=VKxoRqdza6UTnrWhF_fgIA.

13 https://github.com/cleanlab/cleanlab.

14 https://github.com/aub-mind/arabert.

15 https://developer.twitter.com/en/developer-terms/agreement-and-policy.
